# Investigation of the neural basis of expectation-based analgesia in the human brainstem and spinal cord by means of functional magnetic resonance imaging

**DOI:** 10.1016/j.ynpai.2021.100068

**Published:** 2021-07-21

**Authors:** P.W. Stroman, J.M. Powers, G. Ioachim, H.J.M. Warren, K. McNeil

**Affiliations:** a Centre for Neuroscience Studies, Queen’s University, Kingston, Ontario K7L 3N6, Canada; b Department of Biomedical and Molecular Sciences, Queen’s University, Kingston, Ontario K7L 3N6, Canada; c Department of Physics, Queen’s University, Kingston, Ontario K7L 3N6, Canada; d Royal Military College of Canada, Kingston, Ontario K7L 3N6, Canada

**Keywords:** Pain, Functional magnetic resonance imaging, Spinal cord, Brainstem, Expectation

## Abstract

**Purpose:**

The expected intensity of pain resulting from a noxious stimulus has been observed to have a strong influence on the pain that is perceived. The neural basis of pain reduction, as a result of expecting lower pain, was investigated using functional magnetic resonance imaging (fMRI) in the brainstem and spinal cord.

**Methods:**

Functional MRI studies were carried out in a region spanning the brainstem and cervical spinal cord in healthy participants. Participants were familiarized with a noxious heat stimulus and study procedures in advance, and were informed during each trial that either a heat calibrated to produce moderate pain (Base state), or a temperature 1 °C lower (Low state), would be applied to their hand. However, the Base temperature was applied in every trial.

**Results:**

Pain ratings were significantly reduced as a result of expecting lower temperatures. FMRI results demonstrate blood oxygenation-level dependent (BOLD) signal variations in response to participants being informed of the stimulus to expect, in advance of stimulation, and in response to stimulation. Significant coordination of BOLD signals was also detected across specific brainstem and spinal cord regions, with connectivity strengths that varied significantly with the study condition, and with individual pain ratings. The results identify regions that are known to be involved with arousal and autonomic regulation.

**Conclusions:**

Expectation-based analgesia is mediated by descending regulation of spinal cord nociceptive responses. This regulation appears to be related to arousal and autonomic regulation, consistent with the cognitive/affective dimension of pain.

## Highlights

• Expectation of lower pain results in lower perceived pain in healthy humans.
• This expectation analgesia is mediated by descending regulation of the spinal cord.
• Connectivity analyses showed effects of expecting lower pain prior to stimulation.
• Expectation analgesia involves regions linked to arousal and autonomic regulation.

## 1 Introduction

Pain perception is strongly influenced by cognitive factors such as the expected severity or intensity of impending pain, or expected changes in pain (Villemure and Bushnell, 2002, Rhudy et al., 2006, Gollub et al., 2018, Henderson et al., 2020). Expectation effects can also have a strong influence on experimentally-induced pain for research purposes, and on the effectiveness of pain treatments, such as when coupled with an inactive treatment to produce placebo or nocebo effects (Ružić et al., 2017, Evers et al., 2018). Behavioral studies have demonstrated that expectation of higher pain results in increased pain ratings, and expectation of lower pain results in lower pain ratings (De Pascalis et al., 2002, Ružić et al., 2017). Recent studies of the neural processes underlying expectation influences on pain have employed primarily functional magnetic resonance imaging (fMRI) across networks of interconnected regions. These studies have consistently identified the involvement of the insula, anterior cingulate cortex (ACC), regions of the frontal cortex, thalamus, and periaqueductal gray matter (PAG) in mediating effects of expectation on pain perception (Wager et al., 2004, Schenk et al., 2014, Gollub et al., 2018). Differences in blood oxygenation-level dependent (BOLD) responses have also been identified as a result of placebo and nocebo effects in the dorsal horn of the spinal cord (SC) (Eippert et al., 2009, Tinnermann et al., 2017). The involvement of the ACC, PAG, and SC are consistently shown across studies, and suggest that excitatory and inhibitory input to the SC, primarily from brainstem (BS) regions, to influence SC responses to nociceptive signaling (i.e. descending regulation) may contribute to effects of expectation (Millan, 2002, Henderson et al., 2020). These results also indicate that the three main types of expectancy effects (placebo, nocebo, stimulus expectancy) may have features of neural signaling that are in common (Atlas and Wager, 2012).

Effects of expectation, in conjunction with fMRI and connectivity analyses, may provide a powerful means of investigating mechanisms of descending pain regulation in humans. Prior fMRI studies by our group have demonstrated neural processes in the BS and SC that are involved with descending pain regulation, and how BOLD responses and connectivity between regions vary with cognitive and emotional influences (Powers et al., 2018). Building on these results, we have demonstrated that BOLD responses in BS/SC regions include both a reactive component, which is the direct response to a stimulus, and a continuous component, which appears to be related to cognitive, emotional, and possibly autonomic processes that play a role in modulating pain (Stroman et al., 2016, Stroman et al., 2018). While feed-back control also occurs as a reaction to ascending nociceptive signaling, the continuous component of descending regulation appears to regulate SC responses to nociceptive input in relation to cognitive and emotional states (Stroman et al., 2018, Ioachim et al., 2019). For the present study, we therefore hypothesize that expectation-based analgesia (i.e. *stimulus expectancy*) involves descending regulation of SC excitability, and involves the continuous component of pain regulation.

## 2 Materials and methods

### 2.1 Participants

Twenty healthy participants (10 males, 10 females, age 23 ± 3 years) were recruited from the local community. Exclusion criteria included any history of chronic pain or fatigue, major medical or neurological illness, psychological distress, and any contraindications for the MRI environment. Informed consent for all study procedures was obtained prior to data collection, and all participants were debriefed at the end of the study. All procedures were approved by our institutional human research ethics board. Eligibility screening was conducted through an on-line form on our lab web site.

Participants completed questionnaires in order to characterize mood, pain catastrophizing, and sensory and affective dimensions of pain, including the Social Desirability Scale (SDS) (Crowne and Marlowe, 1960), Beck Depression Inventory-II (BDI-II) (Beck et al., 1996), the State/Trait Anxiety Inventory (STAI) (Spielberger, 1970), and the Pain Catastrophizing Scale (Sullivan et al., 1995). The SDS assesses the degree to which a participant is concerned with social approval (which may bias pain ratings). The BDI-II assesses the affective, motivational, cognitive, and somatic symptoms of depression. The STAI measures the transient condition of state anxiety and the long standing condition of trait anxiety. The Pain Catastrophizing Scale reflects how a person deals with pain, such as whether they have a tendency to feel helpless and to magnify the threat value of a pain stimulus.

### 2.2 Quantitative sensory testing (QST) and sham MRI

Quantitative sensory testing (QST) and participant training were carried out in a “sham” mock-up of our MRI system adjacent to the MRI Facility. QST consisted of familiarizing participants with 0–100 numerical pain intensity and unpleasantness rating scales (Vierck et al., 1997, Staud et al., 2001). The scales ranged from verbal descriptors of “no sensation” at 0 to “intolerable pain” or “intolerable unpleasantness” at 100. The difference between pain intensity and unpleasantness was described to the participants. Heat stimuli were applied to the palm of the right hand overlying the thenar eminence (corresponding to the 6th cervical dermatome), by means of an MRI-compatible robotic contact-heat thermal stimulator (RTS-2). This device consists of a 4 cm square aluminum thermode which is retracted into a plastic housing, or is advanced to contact the skin. The timing and duration of contacts, and the thermode temperature, were under precise control by MATLAB software.

For training, participants experienced repeated contacts of the thermode on their hand of 1.5 s duration each, with onsets every 3 s, over a range of temperatures to familiarize them with the stimulus, and with rating their pain for each contact. Following initial familiarization, participants rated the intensity of their sensations to 10 consecutive heat contacts at fixed temperatures of 46 °C, 50 °C, 44 °C and 48 °C with two minutes of rest between sets. This stimulation method has been used in our prior studies to evoke temporal summation of second pain, and it provides a dynamic and robust BOLD response (Bosma et al., 2015, Bosma et al., 2016, Stroman et al., 2016). Participants were asked to verbally rate the pain intensity for each contact, as they felt it. This protocol was used to calibrate the temperature needed to elicit moderate pain (rating of approximately 50), but an upper limit of 52 °C was set to avoid causing damage to the skin. The participant was then positioned in a sham MRI scanner and one run of the fMRI protocol (including playback of recorded MRI sounds) was carried out with the calibrated temperature set to evoke moderate pain. During the sham run, participants were asked to silently rate each contact as they felt it, and to remember only the highest intensity and unpleasantness ratings over the 10 contacts. After the run they were asked to report their peak intensity and unpleasantness ratings. The participant was then informed that fMRI runs would be carried out either with this same temperature (“Base”) for some runs, or 1 °C lower (“Low”) for other runs. Unbeknownst to the participants, the Base temperature was applied in all runs, in order to engage the effects of expectation on the perceived pain.

### 2.3 Experimental design and statistical analysis

#### 2.3.1 Magnetic resonance imaging

Imaging was carried out at 3 T (Siemens Magnetom Trio) with the participant positioned supine, with padding and blankets for comfort and to reduce movement. Posterior head, neck, and spine receiver coils were in place for detecting the MRI signal, and a mirror was positioned so that participants could view a rear-projection screen on which the intensity and unpleasantness rating scales and instructions were displayed.

After initial localizer scans, functional MRI was carried out in a series of ten 4.5-minute long runs with the study conditions (Base or Low) varied in randomized order. Functional MRI data were acquired spanning the cervical SC and BS using a T_2_-weighted half-Fourier single-shot fast spin-echo (HASTE) sequence. This method enables us to avoid using echo-planar imaging (EPI) which causes severe spatial distortions in MR images in the vicinity of the bone and airspaces (Hutton et al., 2002). We have established optimal methods that provide a combination of BOLD contrast and excellent image quality in the BS/SC (Bosma and Stroman, 2014). Imaging parameters used for this study include an echo time (TE) of 76 msec for T_2_-weighting, 9 sagittal slices (no gap), and a repetition time (TR) of 6.75 s per volume, with 1.5 mm × 1.5 mm × 2 mm resolution. Image quality is thus gained at the cost of imaging speed because a repetition time of 6.75 s is required to balance image quality and energy deposition limits.

#### 2.3.2 Functional MRI paradigm

**Fig. 1:**
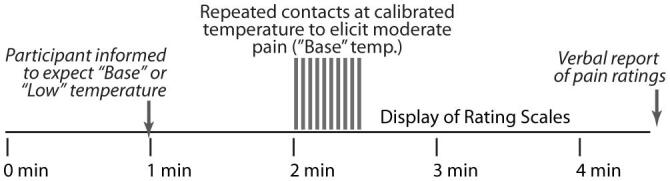
Stimulation paradigm used for functional MRI studies. Participants were informed 1 min after the start of the fMRI acquisition to expect either the “Base” temperature which was calibrated to evoke moderate pain, or a temperature that is 1 °C lower (“Low”). Participants were familiarized with the temperatures and pain rating scales during the training phase. Regardless of the expected temperature, stimulation consisted of repeated contacts with the thermode at the calibrated “Base” temperature.

During each fMRI run, participants were initially informed that a new run was about to begin but they did not know which stimulus temperature to expect (Fig. 1). One minute after the start of the acquisition, participants were informed via the visual display whether the temperature would be the Base or Low temperature, and the temperature was also displayed. One minute later, at the 2-minute mark, 10 heat contacts were applied (onsets every 3 sec, 1.5 sec duration). Again, the thermode temperature was always set to the Base temperature to elicit moderate pain, regardless of how the participant was informed. Thus, any reduction in pain during the Low temperature state was the result of expecting a lower temperature. After the stimulation period, scanning was continued for 2 min, for a total of 4.5 min. After each run, participants verbally reported their peak intensity and unpleasantness ratings.

### 2.4 Data analysis

#### 2.4.1 Analysis of behavioral data

Pain intensity and unpleasantness ratings from each participant were compared between study conditions, and the correlation was computed between the change in pain ratings between conditions (Δpain) and the pain ratings in the Base state. Correlations between pain ratings and questionnaire scores were also investigated.

#### 2.4.2 Functional MRI data preprocessing

Functional images were preprocessed using “spinalfmri9”, a freely-shared software toolbox written in MATLAB (The Mathworks Inc., Natick, MA) that has been used extensively for SC and BS fMRI studies for both image preprocessing and data analysis. Pre-processing steps included conversion to NIfTI format, slice-timing correction, co-registration (i.e. motion correction) using the Medical Image Registration Toolbox (MIRT) (Myronenko and Song, 2010), interpolation to 1 mm^3^ resolution, and then spatial normalization and removal of physiological noise. Spatial smoothing was not applied.

Spatial normalization was guided by a combined anatomical reference image (template) spanning across brain (MNI152 template) and SC regions (PAM50 template), as described by De Leener et al. (2018). Corresponding anatomical region-of-interest maps have also been defined from multiple sources, as described previously (Lang and Bartram, 1982, Talairach and Tournoux, 1988, Millan, 2002, Keren et al., 2009, Naidich et al., 2009, Whitfield-Gabrieli and Nieto-Castanon, 2012, Leijnse and D'Herde, 2016, De Leener et al., 2017, Pauli et al., 2018, Chiang et al., 2019, Liebe et al., 2020, Stroman et al., 2020) (https://identifiers.org/neurovault.collection:3145, www.med.harvard.edu/AANLIB/).

Physiological noise was modeled based on peripheral pulse recordings, bulk motion, and global signal variations in white matter, and removed from the data. Our methods of removing physiological noise have been validated, including comparisons with data from cadavers, and are highly effective (Bosma and Stroman, 2014, Harita and Stroman, 2017).

Finally, the first two volumes of each run were discarded to avoid periods without consistent T_1_-weighting, and the remaining time-series responses for each voxel were converted to a percent signal change from the time-series average.

#### 2.4.3 Functional MRI data analysis

##### 2.4.3.1 Connectivity analyses: structural equation modeling (SEM)

Connectivity between BS/SC regions was investigated by means of SEM (Kline, 2011, Stroman, 2016). Using a predefined anatomical model, BOLD responses in a target region were modelled as a weighted sum of BOLD responses in plausible regions providing input signaling (source regions). For example, if region A is modeled as receiving input signaling from regions B and C, and the BOLD time-series responses in these regions are respectively *S_A_*, *S_B_*, and *S_C_*, then *S_A_* = *β_AB_ S_B_* + *β_AC_ S_C_* + *e_A_*, where *e_A_* is the residual (Stroman, 2016). The linear weighting factors (β-values) were computed for one target region at a time (i.e. region “A” in the equation above) using a gradient-descent method with L1 regularization to avoid over-fitting (Ruder, 2016).

**Fig. 2:**
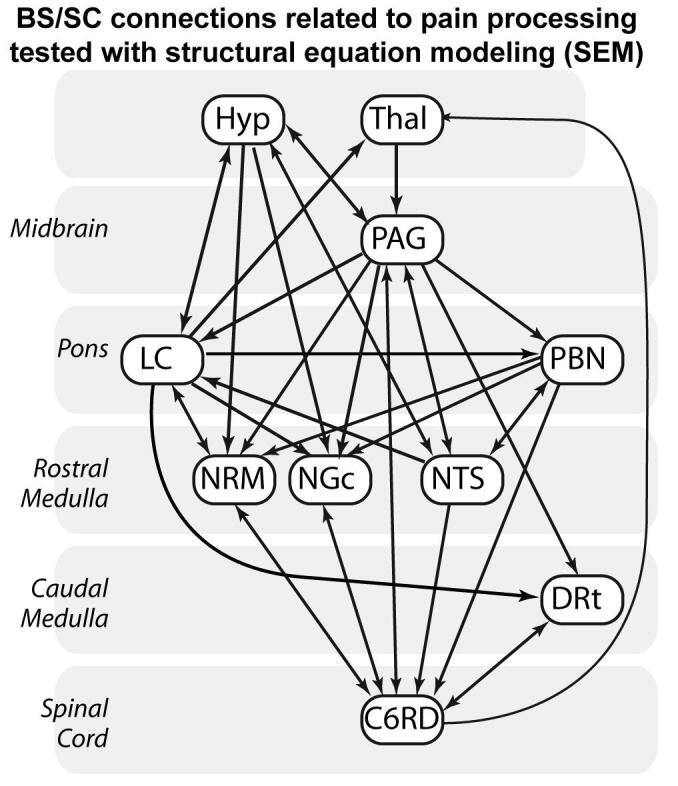
Connections between regions in the brainstem and spinal cord that may be involved with pain processing, and that were included in the model used for structural equation modeling (SEM) analyses. The regions included only those spanned by our data set; the hypothalamus, thalamus (Thal), periaqueductal gray (PAG) region, locus coeruleus (LC), parabrachial nuclei (PBN), nucleus raphe magnus (NRM), nucleus gigantocellularis (NGc), nucleus tractus solitarius (NTS), dorsal reticular nucleus of the medulla (DRt), and the right dorsal region of the spinal cord in the 6th cervical segment (C6 RD).

The network model used for this analysis is based on subcortical anatomical regions and connections that are known to be related to pain processing (Millan, 2002) (Fig. 2). The model includes a total of 38 connections between the thalamus, hypothalamus, PAG, locus coeruleus (LC), parabrachial nucleus (PBN), nucleus raphe magnus (NRM), nucleus gigantocellularis (NGc), nucleus tractus solitarius (NTS), dorsal reticular nucleus of the caudal medulla (DRt), and the right dorsal quadrant of the 6th cervical SC segment (C6RD), corresponding to the region of the right hand where the heat stimulus was applied. As shown in Fig. 2, each region is modeled as having 2 to 6 sources of input signaling.

As it is not practical to apply the SEM method on a voxel-by-voxel basis, it was applied to clusters of voxels within anatomical regions of interest. Regions were first identified in spatially normalized data using the anatomical reference map described above. Voxels within each region were then separated into 5 sub-regions based on their signal time-course properties. This procedure consisted of extracting voxel data from an anatomical region and concatenating the data across all participants/runs. This was done with all data from both study conditions. A k-means clustering method was then used to divide the voxels into clusters with the most similar properties. The resulting cluster definitions were then used to identify the BOLD time-series responses, averaged over the voxels in each sub-region, for each run in each participant.

The SEM procedure was applied to one sub-region of the target region at a time, with combinations of each of the source sub-regions. This method enabled us to identify the combination of sub-regions that yielded the best fits to the measured data. For each of the 38 connections in the model, there were 25 independent combinations of sub-regions (i.e. 5 source sub-regions × 5 target sub-regions for one connection). While a given connection was tested multiple times with different source sub-regions for other connections to the same target, these are not independent comparisons. Statistical characterizations of the resulting β-values (described in Section 2.4.3.2) therefore accounted for 950 independent combinations of network components that were tested.

SEM was applied separately for each participant, in each study condition, in order to enable subsequent analysis of variations in relation to individual responses. The same sub-region definitions were used for all analyses. Analyses were also carried out separately with data extracted from time periods preceding stimulation, and during stimulation, in order to allow for dynamic variations. Specifically, the selected periods spanned 7 volumes, or 47 s. Across 5 runs of the same type in each person, the cluster time-series data thus spanned 35 volumes. These were used to compute between 2 and 6 β-values for each network component, for one combination of sub-regions at a time. The statistical significance of the resulting connectivity values was inferred from the consistency, or from the explained variations in relation to pain responses, across study participants and conditions, as detailed below.

##### 2.4.3.2 Statistical analysis of SEM results

Variations of SEM β values across participants, and between study states, were investigated by means of analyses of covariance (ANCOVAs). An ANCOVA was applied to the β -values from each participant as the dependent variable, with the pain ratings as a continuous independent variable, and the study state (Base or Low) as a discrete independent variable. Significance was inferred at a family-wise error rate controlled p_fwe_ < 0.05 (i.e. uncorrected p < 5.3 × 10^−5^), accounting for the 950 independent connections that were tested. The results of the ANCOVA analysis identify the significance of the main effect of the study state, the unpleasantness or intensity ratings, or interactions between the state and ratings.

Average β-values were also determined for each state, and each connection, in order to characterize the consistent network features and effects of expectation. The significance was estimated based on Student’s T-values, and was inferred at a family-wise error rate corrected, p_fwe_ < 0.05, accounting for the 950 connections that were tested.

##### 2.4.3.3 Analysis of BOLD time-course responses-Bayesian regression

In order to investigate details of BOLD responses in specific regions, we characterized the variations across participants in relation to pain ratings and stimulation temperatures. The purpose of this analysis was to identify the consistent features of BOLD responses in a region. This was applied to the time-course responses for each sub-region, for all participants, using the data that were prepared for the SEM analysis described above. Bayesian regression was applied to each point in the time-series, using unpleasantness ratings and temperatures as independent variables. The ratings and temperatures were first centered so that the average values across participants were equal to zero, and scaled so that the largest differences from the average were equal to one. The data were then fit to approximate the consistent BOLD responses (S_BOLD_) at the average unpleasantness and temperature ratings (S_0_), plus linear estimates of the BOLD variations with unpleasantness ratings (S_p_) and temperature (S_t_) (Stroman et al., 2018):

S_BOLD_ = S_0_ + pain S_p_ + temperature S_t_

The fitting process therefore estimated BOLD response patterns (S_0_) independent of individual differences in sensitivity or the stimulation temperature used, and demonstrates how the BOLD responses varied systematically across participants with different pain responses.

## 3 Results

### 3.1 Behavioral results

**Fig. 3:**
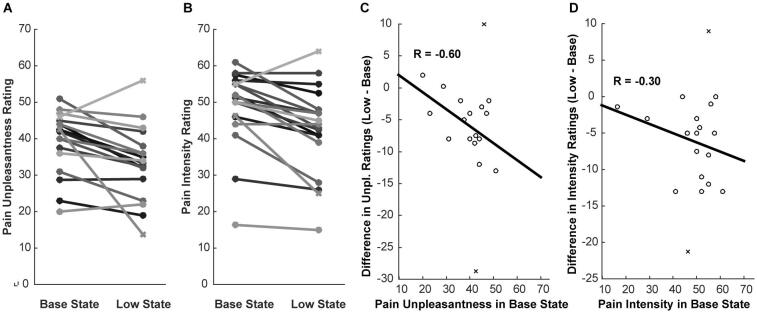
The distributions of A) pain unpleasantness ratings, and B) pain intensity ratings, across participants are shown for the two study conditions (Base and Low). The values for each participant are joined by a line to demonstrate the paired difference. The difference between pain unpleasantness ratings (C) and pain intensity ratings (D) are plotted in relation to the corresponding ratings in the Base condition. The results demonstrate correlations between the change in pain ratings, and the ratings in the Base state. Values plotted with “x” symbols indicate outliers from the group.

Participants reported significant reductions in pain when a 1 °C lower temperature was expected, as compared to the Base temperature. Pain intensity ratings decreased from an average of 48.8 ± 10.4 to 42.6 ± 11.8 (mean ± s.d.) (p = 4.7 × 10^−4^, paired *t*-test), between the Base and Low states, respectively, even though the same temperature was applied in every run. Corresponding ratings of pain unpleasantness decreased from 38.0 ± 9.3 to 32.4 ± 10.1 (p = 3.5 × 10^−3^, paired *t*-test) between the two states. There was a large degree of individual variability within each study state, but an overall consistent trend of lower pain ratings in the Low state (Fig. 3A, B). There was a significant correlation (R = −0.59, Z = 2.12, p = 0.017, n = 18) between the change in unpleasantness ratings between the Base and Low states, and pain unpleasantness ratings in the Base state (Fig. 3C). A weaker correlation (R = −0.30) was also seen between changes in pain intensity ratings between the Base and Low states, and the intensity ratings in the Base state (Fig. 3D). Two participants’ ratings marked in Fig. 3C/D were noted to be outliers in the pain ratings (more than two standard deviations from the group average), and were excluded from further analysis. Moreover, because a stronger correlation was observed with unpleasantness ratings than intensity ratings, we focused the subsequent analyses on the unpleasantness ratings.

### 3.2 Results of questionnaires to assess participant characteristics

Participants scored within normal ranges on questionnaires assessing state and trait anxiety, depression, and pain catastrophizing (Supplementary Table S1). Pain intensity and unpleasantness ratings were also compared with questionnaire scores, and none of the values were significantly correlated (Supplementary Table S2).

### 3.3 Functional MRI results

Significant connections were observed between a number of BS and SC regions in response to the stimulation paradigm, in periods both before and during stimulation. Connections with group average values that were significantly different than zero (p_fwe_ < 0.05), are listed in Table 1. The connections with group average strengths that were significantly different than zero include PAG → PBN in the period before stimulation, and PBN → NGC, and NRM←→C6RD during stimulation. The NTS → C6RD connection strength was also observed to be significantly correlated with pain intensity ratings during stimulation.

**Table 1:** Connectivity values identified with structural equation modeling (SEM), with group average responses that are significantly different than zero (T-test, p_fwe_ < 0.05), or are correlated with pain intensity or unpleasantness ratings (p_fwe_ < 0.05). β-values are listed as the mean ± standard error, and the corresponding statistical values are shown.

Before Stimulation Period	**Expecting Moderate Pain (Base condition)**			
			**β (mean ± s.e.)**	**T**
	Significantly different than zero	PAG → PBN	0.27 ± 0.04	6.296968
			
	**Expecting Lower Pain (Low condition)**			
		no significant connections		
			

During Stimulation Period	**Expecting Moderate Pain (Base condition)**			
			**β (mean ± s.e.)**	**T**
	Significantly different than zero	PBN → NGC	0.26 ± 0.03	7.39
	**Expecting Lower Pain (Low condition)**			
	Significantly different than zero		**β (mean ± s.e.)**	**T**
		NRM → C6RD	−0.12 ± 0.02	−6.63
		C6RD → NRM	−0.21 ± 0.03	−6.26
			**R^2^**	**Z**
	Correlation with intensity ratings	NTS → C6RD	0.67	3.36

**Fig. 4:**
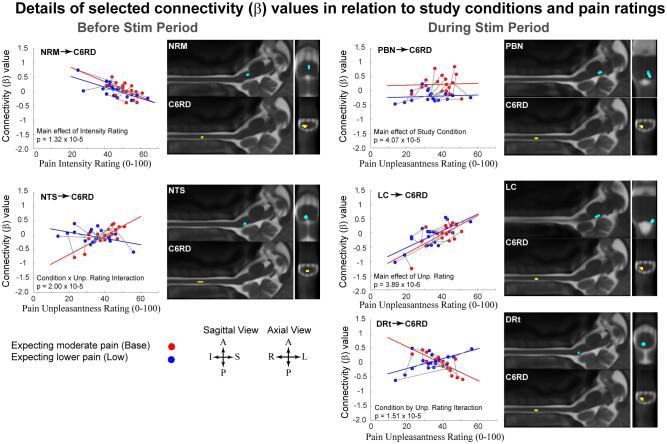
Details of connectivity variations in relation to study conditions and pain unpleasantness ratings, as identified by ANCOVA analyses. Connectivity values for the High temperature condition (expecting moderate pain) are plotted in red, and values for the Low temperature condition (expecting lower pain) are plotted in blue. Lines are drawn (gray) between points in the two conditions for each participant to show the individual differences between conditions. The corresponding anatomical regions are shown in sagittal and axial views, in slices through the centers of each 3D region (interpolated to 1 mm cubic voxels). The results identify known pathways of descending regulation of SC nociceptive responses, via brainstem regions. Connectivity values are shown to vary in relation to pain ratings and study conditions, both before and during the stimulation period. (For interpretation of the references to colour in this figure legend, the reader is referred to the web version of this article.)

**Fig. 5:**
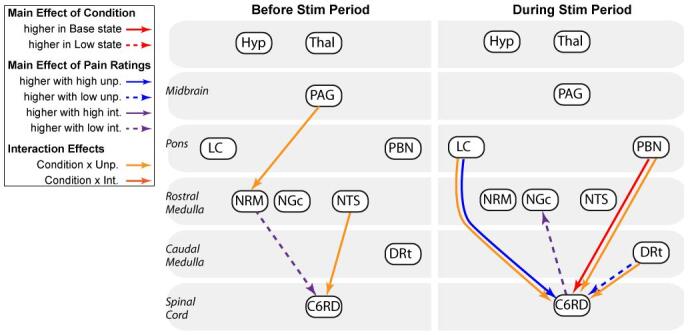
Summary of connections that were identified as being significant by any of the analysis methods. The results show descending pain regulation pathways that vary in relation to the expected stimulus (Base vs Low states), both before and during stimulation, even though the same stimulus was applied in every fMRI run.

Analyses of covariance (ANCOVAs) demonstrated main effects of the study condition (i.e. significant differences between the “Base” and “Low” expectation conditions), main effects of the pain ratings (i.e. significant variations in relation to pain ratings), and significant interaction effects (significant relationships between connectivity values and pain ratings, which differed between study conditions). Details of the ANCOVA results are listed in Table 2, for periods before and during stimulation, and are also shown in Fig. 4 for selected connections. These results identify primarily descending input to the SC right dorsal region (C6RD) from BS regions, although ascending pathways are also shown. The areas with connections to the SC before stimulation include the NTS, and the NRM, which is part of the rostral ventromedial medulla (RVM). During stimulation, connections to the SC (C6RD) were identified from the PBN, LC, and DRt. An ascending connection was also identified from the SC to the NGc, which is also part of the RVM. A PAG → NRM connection is also identified in the period prior to stimulation. The connections identified with these analyses are summarized in Fig. 5.

**Table 2:** Results of analysis of covariance (ANCOVA) applied to connectivity values to investigate dependences on pain unpleasantness and intensity ratings, and study conditions (Base vs Low). . Significant results are shown (p_fwe_ < 0.05).

	Connection	F-value	p-value (p_fwe_ < 0.05, p_uncorrected_ < 5.25 × 10^−5^)
Before Stimulation Period	**Main effect of Intensity Rating**		
	NRM → C6RD	26.43	1.32 × 10^−5^
		
	**Interaction Group × Unpleasantness Rating**		
	NTS → C6RD	24.98	2.00 × 10^−5^
	PAG → NRM	21.76	5.25 × 10^−5^
		

During Stimulation Period	**Main effect of Study Condition (Base vs Low)**		
	PBN → C6RD	22.61	4.07 × 10^−5^(based on unp. ratings)
	PBN → C6RD	25.09	1.95 × 10^−5^(based on int. ratings)
		
	**Main effect of Unpleasantness Rating**		
	LC → C6RD	30.9	3.89 × 10^−6^
	DRt → C6RD	22.05	4.79 × 10^−5^
		
	**Main effect of Intensity Rating**		
	C6RD → NGc	23.94	2.69 × 10^−5^
		
	**Interaction Group × Unpleasantness Rating**		
	PBN → C6RD	34.13	1.70 × 10^−6^
	DRt → C6RD	25.92	1.51 × 10^−5^
	LC → C6RD	23.71	2.88 × 10^−5^

**Fig. 6:**
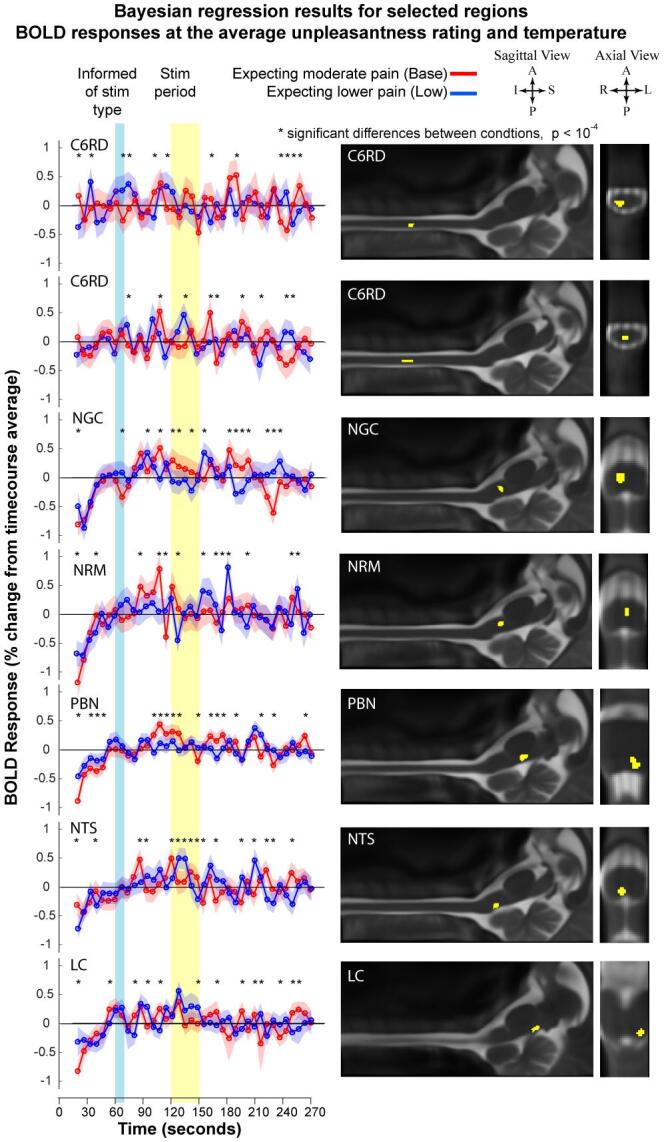
Bayesian regions results for selected regions, showing expected BOLD responses at the average unpleasantness rating and temperature across the group, for each condition. The corresponding anatomical regions are shown in sagittal and axial views (interpolated to 1 mm cubic voxels). BOLD responses when participants expected moderate pain (Base state) are plotted in red, and responses when participants expected the stimulus to be 1 °C lower (Low state) are plotted in blue. The shaded regions indicate the standard errors of the responses at each time point. BOLD variations are demonstrated in response to participants being informed of what to expect (the time period indicated in blue) and in the period shortly after, in each of these regions. Variations are also demonstrated in the period leading up to stimulation. The stimulation period is indicated in yellow. Significant differences between conditions (p < 10^−4^) are indicated by an asterisk (*). BOLD signals during stimulation are demonstrated in almost every region shown to depend on the study condition. BOLD responses are also demonstrated in response to the participants before informed of the stimulus to expect, and in the period preceding the stimulus. (For interpretation of the references to colour in this figure legend, the reader is referred to the web version of this article.)

Bayesian regression results show the consistent features of how BOLD responses varied across participants in relation to different pain ratings and stimulation temperatures, as well as the expected BOLD timecourse responses at the average unpleasantness rating and temperature. Results for selected regions/sub-regions are shown in Fig. 6. In the SC right dorsal region at C6 (C6RD) these results (displayed for two separate C6RD sub-regions) show a pattern of responses to the participants being informed of the type of stimulus to expect, followed by increasing BOLD signal in the time leading up to the stimulation period. The two sub-regions have different BOLD responses during the stimulation period, with one showing increased signal only during the “Base” condition, and the other showing increased signal only during the “Low” condition. In the NGc, the BOLD responses are initially similar during the two conditions, but the response is significantly higher in the Base condition just prior to stimulation, during stimulation, and for a period of time after stimulation. BOLD responses in the NRM show similar trends with signal increases in the period leading up to stimulation, except for a notable increase in signal in the Base condition prior to stimulation. BOLD responses in the PBN are somewhat similar to those in the NGc, as they are significantly higher in the Base condition just prior to stimulation and at the beginning of the stimulation period, and also higher for a period after stimulation. In the NTS an increase in signal occurred after the participants were informed of what to expect in the Base condition, and the signal was higher during stimulation in the Low condition. Finally, BOLD responses in the LC also had increased signal when participants were informed of what to expect in both conditions, and the signal is higher during stimulation in the Low condition.

## 4 Discussion

Having participants expect a lower stimulation temperature consistently resulted in lower pain ratings, compared to when the Base temperature was expected. Averaged across the group, the effect was a roughly 6-point drop in both pain intensity and unpleasantness ratings, or approximately 12–15% of the base ratings. There was a large degree of individual variability observed, but the pain rating changes between states were correlated with pain ratings in the Base state. This indicates that the magnitude of the expectation effect was related to individual traits of pain responses. Moreover, the correlation observed with unpleasantness ratings may reflect their stronger relationship to the cognitive/affective component of pain (Rainville et al., 1999). The magnitude of the expectation effect was not correlated with any of the questionnaire scores that we obtained, including social desirability, anxiety, depression, and pain catastrophizing. Given that the temperature applied to each participant was actually the same for the two study states, the behavioral results confirm that expectation modulation of pain was achieved.

Functional MRI results demonstrated differences in connectivity between a number of regions of the lower brain, BS, and SC between different states of pain expectation (Base vs Low), and in relation to individual pain responses. Moreover, differences in connectivity between states were observed both before and during stimulation. Almost all of the connections identified by means of ANCOVAs as having significant dependences on pain ratings and/or the study state (Base vs Low) involved input signaling to the spinal cord dorsal horn (C6RD). The only exceptions were PAG → NRM in the period before stimulation, and C6RD → NGc during stimulation. Both the NRM and NGc are within the RVM and are known to be involved with descending regulation of SC nociceptive responses (Millan, 2002). The connections identified, as summarized in Fig. 5, thus identify multiple components of descending regulation of the SC that contribute to expectation analgesia.

BOLD responses in a number of regions that were identified by the ANCOVA analysis showed responses to the participants being informed of the stimulus to expect and/or possible anticipation of the stimulus. Responses to being informed are demonstrated in the C6RD region, and appear to be different between the two conditions (Base vs Low), but depends on the sub-region of the spinal cord. Responses at this time are also demonstrated in the PBN and LC. BOLD increases are also shown in the period just prior to stimulation in the SC as well as in the NRM, NGc, and PBN. BOLD responses during stimulation were not consistently observed in all regions, with C6RD sub-regions showing BOLD increases which depended on the study condition, and the NTS and LC showing larger responses in the Low condition. The results demonstrate that these BS and SC regions responded to the participants being informed of what to expect, and to the noxious heat stimulus. The only connection with connectivity values with a significant main effect of the study condition, was PBN → C6RD, and LC and DRt inputs to the SC showed dependences on both the study condition and individual pain responses. Given that the same temperature was applied for each participant in every run, but pain ratings were significantly different between conditions, we can conclude that the PBN, LC, and DRt modulated nociceptive responses in the SC in relation to the pain that was expected. The results also further demonstrate the continuous nature of descending regulation of the SC, as opposed to responses being only in reaction to nociceptive signaling.

While it is expected that other connections contributed to descending modulation of SC responses, and also ascending feed-back from the SC, no other connections reached statistical significance after correcting for multiple comparisons. It is also possible that individual variability is not adequately described by a simple linear relationship between connectivity values and pain ratings. We expect that individual variability is more complex, and involves different strategies of coping with pain responses, and variations in participants’ attention focus during the fMRI runs, as suggested by other studies (Kucyi et al., 2013). The contribution of PBN → C6RD signaling to expectation modulation of pain is the most prominent in our data, and may have been the most consistent across participants. We note that, prior to stimulation, the PAG had significant effects of modulating the NRM, and the NTS input to the SC varied with pain ratings and study conditions (interaction effect). These connections are thus expected to have contributed to variations in descending regulation of SC nociceptive responses as well, but it is unclear if signaling prior to stimulation influenced the subsequent pain response, or if it only reflects the state of pain responsiveness during that period.

During the stimulation period, input signaling to the SC from the LC and DRt varied significantly in relation to pain unpleasantness ratings, and SC input from the PBN varied in relation to the study condition, although all three connections also showed significant interaction effects between the study condition and pain unpleasantness ratings. It appears that the LC and DRt connections are more strongly related to individual differences in pain responses whereas the PBN activity is more closely linked to the differences in study conditions, but all three of these regions appear to have contributed to the observed effect of expectation modulation of pain.

The regions of the RVM contribute to descending pain regulation by modulating the excitability of SC neurons to nociceptive input (Millan, 2002, Ossipov et al., 2010). A number of regions provide input signaling to the RVM including the hypothalamus, PAG, LC, and PBN to influence descending pain regulation, and the NTS, LC, PBN and DRt can also provide direct input signaling to the SC. The present results indicate that pain modulation in relation to expectation of lower pain involves NTS, LC, PBN, and DRt input to the SC, and that the actions or influences are different for each of these regions. The NTS has been shown to be involved with autonomic homeostasis (Craig, 2003), and may be an interface between autonomic and pain functions (Lewis et al., 1987). This region appears to have had a stronger influence in the period prior to stimulation. The link between pain regulation and autonomic function is further reinforced by the role of the PBN, which contributes to integrating autonomic and somatosensory information (Millan, 2002), and this region’s influence was stronger during stimulation. The LC is the major producer of noradrenaline in the central nervous system, is part of the reticular activating system which regulates stress and arousal, and is involved with ascending signaling as well as descending regulation of the SC (Suto et al., 2014, Llorca-Torralba et al., 2016). The DRt has been shown to be pro-nociceptive and forms a feed-back loop with the spinal cord to regulate pain responses. The connections identified by the current study are therefore consistent with known descending regulation and suggest that expectation modulation of pain is related to the state of arousal and autonomic regulation.

When a noxious stimulus is applied to the periphery, nociceptive signaling is relayed from SC neurons to cortical regions, the thalamus, and BS regions (Millan, 2002). Feed-back regulation of SC responses to nociceptive input involves the PAG → RVM → cord pathway, which is modulated by a number of inputs (as described by the model used for SEM analysis, Fig. 2). The resulting complex network of feed-back pathways regulates pain and balances the many influential factors. The anatomy of the autonomic regulating network and afferent feed-back involved with interoception (the sense of the internal state of the body) described by Craig (2003) closely parallels the pain regulating network in the BS. It includes multiple feed-back loops for somato-autonomic reflexes, and includes the NTS and PBN (Sato and Schmidt, 1973, Craig, 1993, Craig, 1995, Craig, 2003). Price and Harkins (Price and Harkins, 1992) summarized the affective dimension of pain as being “the end product of multiple contributing processes, including the pain sensation itself, arousal, autonomic and somatomotor activation, and finally and most critically, cognitive appraisal”. The results of the present study therefore describe a specific effect within these integrated networks, and demonstrate BS/SC pathways that are involved with the influence of expecting a lower stimulus temperature on the level of pain that is produced.

## 5 Conclusions

The results provide evidence that expectation elicits modulation of nociceptive signaling in the SC, in relation to arousal (via the LC), and in relation to autonomic regulation (via the NTS and PBN). We propose that the autonomic link may be related to interoception and its influence on the cognitive appraisal of pain, as opposed to the effect of pain on autonomic responses, because it was observed as input signaling to the SC in the dermatome that was stimulated (i.e. PBN → C6RD), and varied between expectation conditions. The results provide a previously unexplored view of human pain regulation in the BS/SC and how expectation can modulate pain, and add to our overall understanding of healthy human pain processes.

## CRediT authorship contribution statement

**P.W. Stroman:** Conceptualization, Data curation, Formal analysis, Funding acquisition, Methodology, Project administration, Software, Supervision, Visualization, Writing - review & editing. **J.M. Powers:** Conceptualization, Visualization, Writing - review & editing. **G. Ioachim:** Conceptualization, Visualization, Writing - review & editing. **H.J.M. Warren:** Writing - review & editing. **K. McNeil:** Writing - review & editing.

## Declaration of Competing Interest

The authors declare that they have no known competing financial interests or personal relationships that could have appeared to influence the work reported in this paper.

## References

[b0005] Atlas L.Y., Wager T.D. (2012). How expectations shape pain. Neurosci. Lett..

[b0010] Beck A.T., Steer R.A., Ball R., Ranieri W.F. (1996). Comparison of Beck Depression Inventories -IA and -II in psychiatric outpatients. J. Pers. Assess..

[b0015] Bosma R.L., Ameli Mojarad E., Leung L., Pukall C., Staud R., Stroman P.W. (2015). Neural correlates of temporal summation of second pain in the human brainstem and spinal cord. Hum. Brain Mapp..

[b0020] Bosma R.L., Mojarad E.A., Leung L., Pukall C., Staud R., Stroman P.W. (2016). FMRI of spinal and supra-spinal correlates of temporal pain summation in fibromyalgia patients. Hum. Brain Mapp..

[b0025] Bosma R.L., Stroman P.W. (2014). Assessment of data acquisition parameters, and analysis techniques for noise reduction in spinal cord fMRI data. Magn. Reson. Imaging.

[b0030] Chiang M.C., Bowen A., Schier L.A., Tupone D., Uddin O., Heinricher M.M. (2019). Parabrachial complex: a hub for pain and aversion. J. Neurosci..

[b0035] Craig A.D. (1993). Propriospinal input to thoracolumbar sympathetic nuclei from cervical and lumbar lamina I neurons in the cat and the monkey. J. Comp. Neurol..

[b0040] Craig A.D. (1995). Distribution of brainstem projections from spinal lamina I neurons in the cat and the monkey. J. Comp. Neurol..

[b0045] Craig A.D. (2003). Interoception: the sense of the physiological condition of the body. Curr. Opin. Neurobiol..

[b0050] Crowne D.P., Marlowe D. (1960). A new scale of social desirability independent of psychopathology. J. Consult Psychol..

[b0055] De Leener B., Fonov V.S., Collins D.L., Callot V., Stikov N., Cohen-Adad J. (2018). PAM50: Unbiased multimodal template of the brainstem and spinal cord aligned with the ICBM152 space. Neuroimage.

[b0060] De Leener, B., Levy, S., Dupont, S.M., Fonov, V.S., Stikov, N., Louis Collins, D., Callot, V., Cohen-Adad, J., 2017. SCT: Spinal Cord Toolbox, an open-source software for processing spinal cord MRI data. Neuroimage 145(Pt A), 24–43. 10.1016/j.neuroimage.2016.10.009 27720818

[b0065] De Pascalis V., Chiaradia C., Carotenuto E. (2002). The contribution of suggestibility and expectation to placebo analgesia phenomenon in an experimental setting. Pain.

[b0070] Eippert, F., Finsterbusch, J., Bingel, U., Buchel, C., 2009. Direct evidence for spinal cord involvement in placebo analgesia. Science 326(5951), 404. 10.1126/science.1180142 19833962

[b0075] Evers A.W.M., Colloca L., Blease C., Annoni M., Atlas L.Y., Benedetti F., Bingel U., Buchel C., Carvalho C., Colagiuri B., Crum A.J., Enck P., Gaab J., Geers A.L., Howick J., Jensen K.B., Kirsch I., Meissner K., Napadow V., Peerdeman K.J., Raz A., Rief W., Vase L., Wager T.D., Wampold B.E., Weimer K., Wiech K., Kaptchuk T.J., Klinger R., Kelley J.M. (2018). Implications of placebo and nocebo effects for clinical practice: expert consensus. Psychother. Psychosom..

[b0080] Gollub R.L., Kirsch I., Maleki N., Wasan A.D., Edwards R.R., Tu Y., Kaptchuk T.J., Kong J. (2018). A functional neuroimaging study of expectancy effects on pain response in patients with knee osteoarthritis. J. Pain.

[b0085] Harita S., Stroman P.W. (2017). Confirmation of resting-state BOLD fluctuations in the human brainstem and spinal cord after identification and removal of physiological noise. Magn. Reson. Med..

[b0090] Henderson L.A., Di Pietro F., Youssef A.M., Lee S., Tam S., Akhter R., Mills E.P., Murray G.M., Peck C.C., Macey P.M. (2020). Effect of expectation on pain processing: a psychophysics and functional MRI analysis. Front. Neurosci..

[b0095] Hutton C., Bork A., Josephs O., Deichmann R., Ashburner J., Turner R. (2002). Image distortion correction in fMRI: a quantitative evaluation. Neuroimage.

[b0100] Ioachim G., Powers J.M., Stroman P.W. (2019). Comparing coordinated networks across the brainstem and spinal cord in the resting state and altered cognitive state. Brain Connect..

[b0105] Keren N.I., Lozar C.T., Harris K.C., Morgan P.S., Eckert M.A. (2009). In vivo mapping of the human locus coeruleus. Neuroimage.

[b0110] Kline R.B. (2011). Principles and Practice of Structural Equation Modeling.

[b0115] Kucyi A., Salomons T.V., Davis K.D. (2013). Mind wandering away from pain dynamically engages antinociceptive and default mode brain networks. Proc. Natl. Acad. Sci. U.S.A..

[b0120] Lang J., Bartram C.T. (1982). Fila radicularia of the ventral and dorsal radices of the human spinal cord. Gegenbaurs Morphol Jahrb.

[b0125] Leijnse J.N., D'Herde K. (2016). Revisiting the segmental organization of the human spinal cord. J. Anat..

[b0130] Lewis J.W., Baldrighi G., Akil H. (1987). A possible interface between autonomic function and pain control: opioid analgesia and the nucleus tractus solitarius. Brain Res..

[b0135] Liebe T., Kaufmann Jörn, Li M., Skalej M., Wagner G., Walter M. (2020). In vivo anatomical mapping of human locus coeruleus functional connectivity at 3 T MRI. Hum. Brain Mapp..

[b0140] Llorca-Torralba M., Borges G., Neto F., Mico J.A., Berrocoso E. (2016). Noradrenergic Locus Coeruleus pathways in pain modulation. Neuroscience.

[b0145] Millan M.J. (2002). Descending control of pain. Prog. Neurobiol..

[b0150] Myronenko A., Song X. (2010). Intensity-based image registration by minimizing residual complexity. IEEE Trans. Med. Imaging.

[b0155] Naidich T.P., Duvernoy H.M., Delman B.N., Sorensen A.G., Kollias S.S., Haacke E.M. (2009). Internal Architecture of the Brain Stem with Key Axial Sections.

[b0160] Ossipov M.H., Dussor G.O., Porreca F. (2010). Central modulation of pain. J. Clin. Invest..

[b0165] Pauli W.M., Nili A.N., Tyszka J.M. (2018). A high-resolution probabilistic in vivo atlas of human subcortical brain nuclei. Sci. Data.

[b0170] Powers J., Ioachim G., Stroman P. (2018). Ten key insights into the use of spinal cord fMRI. Brain Sci..

[b0175] Price D.D., Harkins S.W. (1992). The affective-motivational dimension of pain. A two-stage model. APS J..

[b0180] Rainville P., Carrier B., Hofbauer R.K., Bushnell C.M., Duncan G.H. (1999). Dissociation of sensory and affective dimensions of pain using hypnotic modulation. Pain.

[b0185] Rhudy J.L., Williams A.E., McCabe K.M., Rambo P.L., Russell J.L. (2006). Emotional modulation of spinal nociception and pain: the impact of predictable noxious stimulation. Pain.

[b0190] Ruder, S., 2016. An overview of gradient descent optimization algorithms. arXiv preprint arXiv:1609.04747.

[b0195] Ruzic V., Ivanec D., Modic Stanke K. (2017). Effect of expectation on pain assessment of lower- and higher-intensity stimuli. Scand. J. Pain.

[b0200] Sato A., Schmidt R.F. (1973). Somatosympathetic reflexes: afferent fibers, central pathways, discharge characteristics. Physiol. Rev..

[b0205] Schenk L.A., Sprenger C., Geuter S., Buchel C. (2014). Expectation requires treatment to boost pain relief: an fMRI study. Pain.

[b0210] Spielberger, C.D., 1970. State-Trait Anxiety Inventory.

[b0215] Staud R., Vierck C.J., Cannon R.L., Mauderli A.P., Price D.D. (2001). Abnormal sensitization and temporal summation of second pain (wind-up) in patients with fibromyalgia syndrome. Pain.

[b0220] Stroman P.W. (2016). Validation of structural equation modeling methods for functional MRI data acquired in the human brainstem and spinal cord. Crit. Rev. Biomed. Eng..

[b0225] Stroman P.W., Bosma R.L., Cotoi A.I., Leung R.H., Kornelsen J., Lawrence-Dewar J.M., Pukall C.F., Staud R., Biagini G. (2016). Continuous descending modulation of the spinal cord revealed by functional MRI. PLoS ONE.

[b0230] Stroman P.W., Ioachim G., Powers J.M., Staud R., Pukall C. (2018). Pain processing in the human brainstem and spinal cord before, during, and after the application of noxious heat stimuli. Pain.

[b0235] Stroman P.W., Warren H.J.M., Ioachim G., Powers J.M., McNeil K., Bergsland N. (2020). A comparison of the effectiveness of functional MRI analysis methods for pain research: the new normal. PLoS ONE.

[b0240] Sullivan M.J.L., Bishop S.R., Pivik J. (1995). The pain catastrophizing scale: development and validation. Psychol. Assess..

[b0245] Suto T., Eisenach J.C., Hayashida K. (2014). Peripheral nerve injury and gabapentin, but not their combination, impair attentional behavior via direct effects on noradrenergic signaling in the brain. Pain.

[b0250] Talairach J., Tournoux P. (1988). Co-planar stereotaxic atlas of the human brain.

[b0255] Tinnermann A., Geuter S., Sprenger C., Finsterbusch J., Büchel C. (2017). Interactions between brain and spinal cord mediate value effects in nocebo hyperalgesia. Science.

[b0260] Vierck C.J., Cannon R.L., Fry G., Maixner W., Whitsel B.L. (1997). Characteristics of temporal summation of second pain sensations elicited by brief contact of glabrous skin by a preheated thermode. J. Neurophysiol..

[b0265] Villemure C., Bushnell M.C. (2002). Cognitive modulation of pain: how do attention and emotion influence pain processing?. Pain.

[b0270] Wager T.D., Rilling J.K., Smith E.E., Sokolik A., Casey K.L., Davidson R.J., Kosslyn S.M., Rose R.M., Cohen J.D. (2004). Placebo-induced changes in FMRI in the anticipation and experience of pain. Science.

[b0275] Whitfield-Gabrieli S., Nieto-Castanon A. (2012). Conn: a functional connectivity toolbox for correlated and anticorrelated brain networks. Brain Connect..

