# Supplementary material for: Investigation of the neural basis of expectation-based analgesia in the human brainstem and spinal cord by means of functional magnetic resonance imaging
Source: Neurobiol Pain. 2021 Jul 21;10:100068. doi: 10.1016/j.ynpai.2021.100068 (PMC8333346; doi:10.1016/j.ynpai.2021.100068)
Supplement: Supplementary data 1 [file mmc1.docx]

| **Questionnaire Scores** | | | | | | | | |
| --- | --- | --- | --- | --- | --- | --- | --- | --- |
|  | **STA** | | **SDS** | **BDI** | **Pain Catastrophizing** | | | |
|  | **State** | **Trait** |  |  | **Total** | **Rum.** | **Magn.** | **Help.** |
|  | 32 ± 7  (42%) | 35 ±7  (51%) | 17 ± 6  (Average) | 7 ± 6  (Normal) | 13 ± 8  (32%) | 5 ± 3  (34%) | 3 ± 2  (52%) | 4 ± 4  (30%) |
| **Correlation, R, between questionnaire scores and intensity or unpleasantness ratings:** | | | | | | | | |
| **Intensity** | 0.06 | -0.04 | 0.07 | 0.07 | 0.18 | 0.21 | 0.16 | 0.14 |
| **Unpl.** | -0.11 | -0.03 | 0.03 | 0.03 | 0.16 | 0.09 | 0.21 | 0.16 |

**Table S1**: Results of questionnaires to characterize participants’ personality traits in relation to pain processing. STA: Anxiety State/Trait Assessment, SDS: Social Desirability Scale, BDI: Beck Depression Inventory, and Pain Catastrophizing sub-domains of Rumination (Rum.), Magnification (Magn.) and Helplessness (Help.). Average values and percentiles within normal distributions are indicated where available, or the assessment range is indicated. Correlation R-values between questionnaire scores and each of pain intensity and unpleasantness (Unpl.) are also listed.

| **Correlation between:** | **Effect size (ΔIntensity Rating)** | | |  | **Effect size (ΔUnpleasantness Rating)** | | |
| --- | --- | --- | --- | --- | --- | --- | --- |
|  | **R** | **Z-value** | **p-value** |  | **R** | **Z-value** | **p-value** |
| **STA-Y1** | -0.14 | 0.54 | 0.29 |  | -0.17 | 0.68 | 0.25 |
| **STA-Y2** | -0.05 | 0.21 | 0.42 |  | -0.085 | 0.33 | 0.37 |
| **SD** | 0.03 | 0.11 | 0.45 |  | -0.064 | 0.25 | 0.40 |
| **BDI** | 0.17 | 0.67 | 0.25 |  | -0.073 | 0.28 | 0.39 |
| **PCS total** | -0.06 | 0.25 | 0.40 |  | -0.34 | 1.36 | 0.088 |
| **PCS 1** | -0.12 | 0.48 | 0.32 |  | -0.36 | 1.47 | 0.071 |
| **PCS 2** | 0.21 | 0.84 | 0.20 |  | -0.17 | 0.67 | 0.25 |
| **PCS 3** | -0.18 | 0.69 | 0.24 |  | -0.34 | 1.39 | 0.082 |
|  |  |  |  |  |  |  |  |
| **Base Unp. Rating** |  |  |  |  | -0.59 | 2.63 | 0.0043 |
| **Base Intensity Rating** | -0.30 | 1.19 | 0.12 |  |  |  |  |

**Table S2:** Correlation values between questionnaire scores, and pain intensity and unpleasantness ratings. These values are based on 18 participants, with the two outliers identified in Figure 3 excluded.
